# Synopsis of a Lecture on Syphilis before the Classes in the Dental College of the University of Michigan

**Published:** 1892-06

**Authors:** C. G. Darling

**Affiliations:** Clinical Lecturer on Oral Pathalogy and Surgery in the College of Dental Surgery


### THE DENTAL REGISTER.

VOL. XLVI.] JUNE, 1892. [No. 6.

### COMMUNICATIONS.

### Synopsis of a Lecture on Syphilis Before the Classes in the Dental College of the University of Michigan.

BY C. G. DARLING, M. D.

Clinical Lecturer on Oral Pathology and Surgery in the College of Dental Surgery.

Thousands of volumes have been written on the subject of syphilis, enough, it would seem, to leave us without a doubt concerning its origin, course and treatment.

It has been no small task to select from the mass of literature available the few points about syphilis which are essential to you, that you may understand its course and nature, as you will find it in your practice. We must, first of all, put away the idea that syphilis is essentially and only a venereal disease, and remember the fact, that the innocent may suffer from it as well as the guilty.

Syphilis, says a recent author, interests everybody; in the first place, those who have it, a very appreciative audience, and then those who are not affected by it, that is to say those who run the risk of acquiring it. This disease may be said to precede all written history, and strange it may appear that the record has been left upon the teeth of human beings who lived and died ages ago.

The imprints of hereditary syphilis upon the teeth are so lasting and characteristic that the original appearance may be preserved and read after centuries have elapsed. Those who have taken the trouble to study prehistoric bones say that the history of syphilis is written in every country upon the earth.

In 1887 there were, in the Musee Boca, four skulls bearing undoubted marks of syphilis. All of the skulls are of undoubted antiquity. Suffice it to say, that these relics teach us that syphilis existed in Peru before America was discovered, and the disease was common in that country, for many children's skulls bear evidence of it. Research shows that it was no uncommon disease among the North American Indians before the discovery of America by Columbus, though it is improbable that it was carried to Europe from this country, as some would have us believe, for at one time it was known in European countries as the "American Evil."

When you have been engaged in your work of caring for the teeth, have you ever thought there might be written upon them such pages of history?

Parrot says: "these proofs are superior in certainty to those derived from written documents, and from them we learn that syphilis is probably the most ancient disease of man." Opponents of this theory say that other lesions of the bones and teeth exist than those of syphilis, and that the disease may have undergone many changes since the early period from which we think we can read it. Its written history, which dates back many centuries, shows no change unless it be such modifications as we can show in individual cases by well-directed treatment.

The earliest writings of the Chinese contain a fair history of the disease, which, if true, would indicate that the disease was known in that country two thousand years ago.

Egypt claims the distinction of possessing a fair amount of the disease fourteen centuries B. C.; while Japan has a record written in the ninth century.

The Bible is said to abound with many vague references to the disease, although no true or full description is given. Whatever may have been its history in ancient times does not concern us, but it suddenly came into prominence as an epidemic in 1492, when it was spoken of in France as the "Malady of Naples," because the French soldiers were supposed to have brought it from that place when they returned from one of their invasions. In a few years it was found to exist in all countries of Europe, pervading every rank of society. Its source was not recognized at first, but was attributed to the evil influence of the stars; all writers of the time joined in saying they had never seen anything like it.

During the centuries which followed, all venereal diseases were classed together without distinction, and it was not until 1852 that they were strictly classified, and syphilis received the proper consideration which it demands as a disease.

WHAT IS SYPHILIS?

Syphilis is an infectious, constitutional disease, probably due to a micro-organism, which enters the system by the blood and lymphatics, pursuing a chronic course.

It attacks, primarily, the connective tissue, but no tissue in the body is exempt from its ravages, and no organ is so strong that it may not be invaded by this disease.

It is not necessarily a venereal disease; all that is required to infect the healthy individual is to place some of the blood, discharge, or secretions containing the virus upon an abraded surface, or a surface capable of absorption, and the work is done.

The conclusions that syphilis depends on a micro-organism for its transmission have not been positively proven, though in the year 1884 Lustgarten found a bacillus in an initial lesion and in a gumma. He described them as "slightly-curved rods in the interior of nucleated cells." While the disease is usually contracted by persons holding improper relations, there is no person who does not at some time run the risk of accidental infection. The point where the virus is planted develops in time certain peculiar phenomena. This point bears the name of initial lesion, because it is the first marked indication that the bearer has been infected. The secretion from the initial lesion is highly contagious, and is the most common source from which the virus is derived; though secretions from secondary lesions, the blood and lymph, during the secondary stage, are also contagious, while the tears, milk, sweat and saliva are not contagious unless mixed with the blood or some of the secretions.

The infection is usually direct, commonly by cohabitation, while mouth-to-mouth infection, the result of kissing, is more frequent than would be supposed.

Newmann published a carefully prepared report of cases, where the primary lesion was in other locations than on the genital organs. Eighty-four were reported, of which forty-six were on the lips, some were on the tonsils, others were on the tongue and in the pharynx.

Taylor, in a recent article, mentions two cases occurring in the practice of physicians who contracted it in examination of a body soon after death.

Another found chancres on the scalp conveyed by using a copper comb which was borrowed from a syphilitic friend.

Brousse saw a case where there were seven chancres on the face ; these were doubtless caused by using a razor which had recently been used upon a syphilitic individual.

Primary sores on the lips or within the mouth may be caused by transferring the disease by cigars where the maker, a syphilitic, has moistened them with saliva that he may nicely turn the end ; it may also be conveyed by pipes which are passed from mouth to mouth, tooth-brushes, chewing-gum, whistles, drinking vessels that are in common use, razors, towels, by operations and instruments. Glass-blowers, by using the same pipe in common, occasionally become infected. It matters not the source of infection, whether from primary or secondary lesions, or where the primary lesion may be located, the course and duration are essentially the same.

Dr. Bulkley has recently reported some interesting cases of the unusual ways in which syphilis may be contracted. Out of fifteen hundred cases seen by him, sixty-five had the primary lesion at other points than on the genitals. Thirty-four of these were males and thirty-one were females. In thirty cases the chancre was on the lips, in seven on the fingers (the dentist will please recall this when he is examining the mouth of his many patients), six on the breast, six on the tonsil, five on the tongue, three on the cheek, two on the chin, one on the ear, one on the forearm and one on the hand. He further states that forty per cent. of his female patients had innocently contracted the disease. He found one case where the disease was contracted by wearing a bathing-suit which retained the virus from the person who had previously worn it.

Bedding, canes, toilet articles, and even opera-glasses have been known to convey the disease. Another, where the chancre was on the tongue, contracted the disease by holding pins in her mouth. The patient was engaged in a business (fitting garments) where a number of pins were used, and formed the uncleanly habit of putting them in her mouth.

Physicians and dentists may convey it from one person to another by the caustic holder, or by instruments where they are not properly disinfected. Biting and tooth wounds may convey the disease. The largest chancre ever seen was on the ear of a man who had been bitten in a fight. Pinching and scratching may also be added to the ways of infection. The nursing of infants where the breast has a syphilitic lesion becomes a source of infection to the child also. A mother nursed two children, her own and a foundling, giving to each a breast, in due time the foundling died from a well-defined hereditary syphilis; she then gave her child both breasts; in time the parent and child both developed syphilis. Numerous are the instances where physicians and dentists have contracted the disease while attending to their professional duties. One case is mentioned of a lady who contracted the disease while dressing the eye of a friend who had syphilis. Rubbing the eyes, picking the nose, or carrying soiled fingers to the lips, may be sufficient to produce chancre and all of its followers. Physicians or dentists may contract it while examining the mouth and throat of syphilitic patients; the patient coughs, projecting some of the virus upon the face, it may fall upon the lip or eye and begin its lasting course.

In Russia a "female pretender" infected a large number of persons by removing foreign bodies from the eye by the tip of the tongue; on the tip of her tongue was a well-developed chancre.

Thus we find everywhere unseen danger, the only wonder we can express is that infection is not more common. You must be ever on the alert, not only to save your patients from infection, but to save yourself, your family and future generations..

The course of syphilis, for the convenience of study, may be divided into three stages: the primary, secondary and tertiary, each period being characterized by well-marked symptoms; though the change from one to the other is so gradual that it can hardly be said that one period has ended and another begun, the stages not being sharply defined. The first stage has two well-defined periods: the period of incubation—from the time of infection to the appearance of the chancre; the second period extends from the appearance of the primary lesion, or chancre, to the development of the constitutional conditions. The lesions occurring in the primary stage are, the initial lesion and induration of the lymphatic glands. The secondary stage is one of constitutional manifestation, and is frequently ushered in with fever. During this stage there will be various lesions of the skin and mucous membrane, the eyes and lymphatics, particularly the latter, will be involved, superficial ulcers will appear in the mouth and on the tonsil, though the patient is scarcely aware that his throat is sore. The duration of this period will depend much upon the habits and life of the patient and the thoroughness with which he carries on the properly-directed treatment. The tertiary stage may be entirely avoided or never develop if the patient carefully conducts his life and follows the improved methods of treatment during the early stages. When allowed to pursue its course the disease may develop gummata, and ulcerative lesions, destructive disease of the bones and various affections of the nervous system.

The period of incubation is variable. According to the statistics of Diday, it may vary from twenty-five days to one hundred and twenty-five days, the average period being forty-five days. It never occurs later than six months, rarely it happens that a case is reported with a period of incubation lasting ninety days. During this period no sign or symptom betrays the presence of the disease, but soon a change takes place at the point where the virus penetrated, and only at that point. The tissues at this point pass through a series of changes and in time return to their natural condition.

The point is called the initial lesion and for a time is the only expression of the disease. The first lesion of syphilis is called chancre, sometimes called primary syphilitic ulcer, inital sclerosis or Hunterian chancre.

Whether infection be derived from chancre or a secondary lesion, the primary lesion is always the same and may appear upon any part of the body that is exposed to the virus.

There are four recognized forms or appearances which the initial lesion may present: First, erosion; second, the silvery spot; third, the dry papule; fourth, follicular chancre.

Du Castel states that he twice saw the development of chancre. In one case it appeared as a small red spot, which after several hours turned into a papule. The second became a papule ten hours after its first appearance. The small red spot begins as an erosion, the epithelium is raised and a serous secretion takes place beneath it. Sometimes the epithelium is raised and secretion takes place without a redness. The lesion increases slowly, presenting the appearance known as the "silvery spot," so typical of this form of chancre. This secretion disappears, and when it passes away rapidly, either by exposure or absorption, the surface left may be flat or convex, of a brown or brownish-red color; this forms the dry papule. The follicular variety is not so common as the other forms, is pinkish and elevated; as it increases in size it becomes red in color; it does not require a long time for the lesion to develop into a superficial erosion. The epithelium is destroyed, the floor slightly eroded, while the walls slope gradually, not being very clearly cut; it is usually round or oval, but may assume any shape; it may vary greatly in size from a line to a full inch in diameter when well hardened and fully developed. The secretion at first is serous until the sore is irritated or infected with pus microbes, then suppuration begins. Induration is a very important element in the development of chancre, and begins usually in the first week, not later than two or three weeks; this begins first in the mucous membrane surrounding the sore, and gradually extends to the cellular tissue beneath.

The induration is slight at first, but continues to increase until the chancre begins to heal, when it gradually disappears. The change is probably due to the formation of granulation tissue, though the inflammation is never acute, and is gradually removed by absorption.

Chancres on mucous surfaces rarely leave a scar, while those on the skin remain for years, a shining mark with pigmented edges. Much of the scar depends on suppuration and the destruction of tissue.

The neighboring lymphatic glands become indurated, but the skin covering them does not change color. These glands do not become united, are hard, movable and painless. They rarely suppurate unless they are injured. Only one gland may be affected, or a chain of glands may become indurated; they gradually resume their normal condition after the disappearance of the chancre. When chancre is on the lip or chin, the submaxillary lymphatic is enlarged. When chancre is on the tongue, search for enlargement of the subhyoid glands. Chancre on the eyelid is accompanied by enlargement of the peri auricular glands. Chancre on the finger or forearm by enlargement of the epitrochlear glands, the axillary glands will also show some enlargement; these glands will be enlarged when the chancre is on the breast. Chancre is usually solitary, but more than one may appear if more than one point is inoculated at the same time. When chancre appears on the lip, it is usually considered a trifling lesion; it may first appear as a small crack but gradually grows, becomes raised, and may be crusted over by the dried secretions; there is lack of pain, but after ten or fifteen days the swelling of the submaxillary glands adds weight to the diagnosis.

